# Enhancing Pediatric Obesity Management through Quality Improvement: A Hybrid Approach to Intensive Health Behavior and Lifestyle Treatment in Primary Care

**DOI:** 10.1097/pq9.0000000000000892

**Published:** 2026-07-28

**Authors:** Emily T. Skrocki, Cristian Sarabia, Shengh Xiong, Ayelet Talmi

**Affiliations:** From the * Children’s Hospital Colorado, Aurora, Colo.; † College of Nursing, University of Colorado, Anschutz Medical Campus, Aurora, Colo.; ‡ Division of Child and Adolescents, Department of Psychiatry, University of Colorado School of Medicine, Aurora, Colo.

## Abstract

**Introduction:**

Intensive health behavior and lifestyle treatment (IHBLT) programs can improve weight and reduce comorbidities for children with obesity, but implementation in primary care remains limited. Baseline performance at our institution showed low referral volume, limited participation, and insufficient treatment intensity, indicating gaps in program reach and adoption.

**Methods:**

Utilizing the model for improvement, we led a quality improvement initiative in a large urban academic primary care clinic to increase IHBLT referrals, participation, and treatment intensity. Sequential plan-do-study-act cycles tested provider education, standardized and culturally tailored materials, a hybrid in-person-and-virtual program structure, and engagement strategies. We tracked three process measures: referrals, participation, and program intensity. We tracked outcomes using individuals-moving-range (X-mR) statistical process control charts.

**Results:**

Statistical process control analysis demonstrated that program participation and intervention intensity met Provost criteria for a sustained shift, whereas referral volume demonstrated primarily common-cause variation. Improvements in participation and intervention intensity were maintained over time, with centerlines recalculated to reflect updated process performance. Moving range charts indicated stable variation across all measures.

**Conclusions:**

An iterative, context-responsive system redesign improved the delivery of evidence-based pediatric obesity treatment in our primary care setting. The hybrid IHBLT model, combining in-person visits and technology-supported touchpoints, was associated with sustained gains in participation and treatment intensity, whereas referral volume remained stable. This pragmatic approach leverages existing resources and offers a feasible pathway for primary care clinics seeking to implement guideline-concordant IHBLT while addressing common logistical and equity-related barriers.

## INTRODUCTION

Pediatric obesity is a common chronic condition with substantial long-term health risks. Major professional and public health organizations, including the American Academy of Pediatrics, the US Preventive Services Task Force, and the Centers for Disease Control and Prevention, recommend structured nutrition and physical activity interventions as part of comprehensive obesity care.^1–6^ Intensive health behavior and lifestyle treatment (IHBLT) programs operationalize these recommendations through family-centered behavioral counseling, health education, and skill building. Despite strong evidence and a 2023 American Academy of Pediatrics clinical practice guideline recommending IHBLT for all children with obesity, implementation in primary care remains limited.^1–6^

### Available Knowledge

A robust body of evidence supports multicomponent lifestyle interventions that integrate nutrition counseling, physical activity promotion, and behavioral strategies to improve body mass index (BMI) and related health outcomes in children and adolescents.^1–4,6,7^ Evidence also highlights the importance of treatment dose; programs delivering at least 26 contact hours are more likely to achieve sustained reductions in BMI and meaningful behavior change.^2,5^ However, many clinical settings struggle to deliver this level of intensity because of workflow constraints, competing demands, and limited resources.

Emerging evidence suggests that virtual and technology-supported approaches can augment in-person care and improve dietary behaviors, physical activity, and weight-related outcomes.^2,8–10^ The Community Preventive Services Task Force endorses technology-supported counseling as an evidence-based strategy, and systematic reviews report small-to-moderate benefits when digital components supplement face-to-face care.^8–10^ Together, these findings suggest that hybrid delivery models may help primary care clinics increase the reach and intensity of IHBLT programs while addressing common logistical barriers. Despite this evidence, integrating such models into routine primary care workflows remains challenging.

Across recent pediatric obesity quality improvement initiatives, effective strategies consistently fall into 2 actionable categories: improving the reliability of provider actions during routine care and redesigning care delivery to reduce barriers to follow-up and sustained engagement. Interventions that focus on provider behavior show that embedding expectations into routine workflows matters. For example, Kharofa et al^11^ demonstrated that pairing brief provider education with electronic health record templates, discharge-order defaults, and performance feedback increased appropriate prescribing of weight management follow-up in primary care, but persistent no-show rates highlighted the limits of ordering alone when patient-engagement barriers remain. Similar projects addressing screening, medication dosing, and comorbidity management reinforce the idea that standardized processes and decision support can improve guideline-concordant care, yet often stop short of ensuring that care is delivered with sufficient intensity.^12–14^ In contrast, initiatives that redesign care delivery to address access barriers show stronger effects on visit completion and continuity. Vallabhan et al^15^ used the model for improvement to implement telemedicine in a pediatric weight management clinic, achieving sustained increases in completed visits, markedly lower no-show rates compared with in-person care, and improved financial sustainability. Together, these studies suggest that clinics seeking to strengthen weight management programs should pair reliable, workflow-embedded provider actions with delivery models that reduce logistical barriers and support ongoing engagement, thereby increasing the likelihood that evidence-based care is not only prescribed but received.

### Local Problem

Before this initiative, our primary care clinic did not use a standardized IHBLT model. Lifestyle counseling varied by provider, lacked structured follow-up, and fell well below recommended treatment intensity. Baseline data from April to July 2023 showed a mean of 8.5 referrals per month, 5.5 participating families per month, and only 1.1 contact hours per participant per month. This level of contact was unlikely to support sustained behavior change or meaningful weight-related improvement.

### Rationale

Given the established relationship between treatment dose and clinical effectiveness, we theorized that a hybrid model combining in-person visits with technology-supported touchpoints could expand program reach, strengthen family engagement, and increase treatment intensity. We further anticipated that standardizing the curriculum and tailoring materials for language and health literacy would improve understanding and trust, thereby reducing attrition. By embedding this hybrid model into existing workflows and leveraging the electronic medical record and patient portal, we sought to implement a pragmatic and sustainable approach to delivering guideline-concordant obesity care in a busy primary care setting.

### Specific Aims

We designed a quality improvement project to strengthen the reach, engagement, and adoption of IHBLT in our primary care clinic over 12 months. We aimed to achieve a 50% improvement across three process measures: increasing mean monthly referrals to approximately 13 referrals per month; increasing mean monthly participation to approximately 8 encounters per month; and increasing mean program intensity to approximately 1.5 contact hours per participant per month.

## METHODS

### Context

This project took place in a large, urban, academic primary care clinic that provides more than 30,000 pediatric visits each year to a culturally diverse population, with roughly 85% of patients publicly insured. Many families experience social drivers of health inequity, including food insecurity, transportation challenges, limited health literacy, and language barriers, which affect their ability to participate in chronic disease management. Care is delivered through a high-volume, team-based model staffed by pediatricians, pediatric nurse practitioners, physician assistants, behavioral health clinicians, nursing care coordinators, and support staff. Despite organizational emphasis on preventive care, chronic disease management, and health equity, the clinic’s fast-paced clinical workflows and limited protected time for counseling contributed to inconsistent, low-intensity lifestyle-management visits. Referral processes were inconsistent, and limited data infrastructure hindered reliable tracking of participation and outcomes. The clinic’s strategic priorities, including strengthening family-centered care and expanding behavioral health integration, created an environment that was receptive to structured IHBLT implementation and system redesign.

### Interventions

**Fig. 1.**
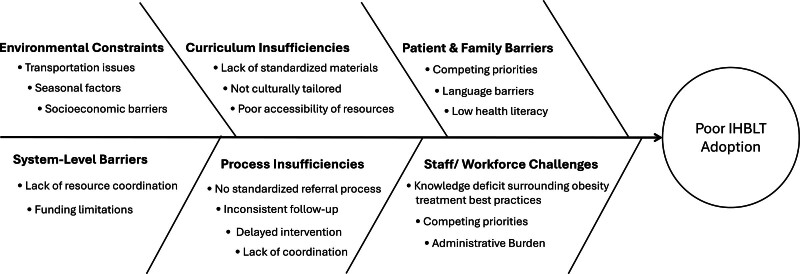
Fishbone diagram of barriers to Intensive Health Behavior and Lifestyle Treatment (IHBLT) implementation. Cause‑and‑effect (fishbone) diagram illustrating multilevel barriers identified through Gemba walks and team review. Barriers were categorized across patient and family factors, environmental constraints, process insufficiencies, system‑level barriers, staff and workforce challenges, and curriculum insufficiencies. These findings informed the prioritization of interventions tested during sequential PDSA cycles.

We assembled a multidisciplinary team of pediatric clinicians, behavioral health specialists, public health practitioners, and a medical librarian with expertise in health literacy. Beginning in August 2023, the team used the Model for Improvement^16^ to guide iterative testing of change. We conducted Gemba walks^17^ to observe workflows, developed a cause-and-effect fishbone diagram (Fig. 1) to map barriers across patient, staff, and system levels, and used these findings to prioritize intervention targets. Table 1 summarizes the plan-do-study-act (PDSA) cycles conducted during this initiative.

**Table 1.** Summary of PDSA Cycles and Associated SPC Centerline Shifts

PDSA Cycle	Plan	Do	Study	Act
PDSA cycle 1	Identify gaps in formal IHBLT structure; clarify program components; develop culturally and linguistically appropriate materials; introduce virtual touchpoints.	Standardized curriculum across behavior, nutrition, and physical activity; created and refined educational handouts; translated materials into Spanish; launched hybrid model with monthly in-person visits and weekly portal messaging; delivered provider education on referrals and workflow.	SPC charts showed no improvement from baseline during this cycle. Referrals declined early with a special-cause low point and did not demonstrate a centerline shift. Participation remained low and variable without sustained change. Treatment intensity stayed near baseline with no evidence of special-cause improvement.	Identified ongoing staff uncertainty regarding referral criteria and scheduling, and family challenges with portal use. Planned enhanced staff education and improved onboarding to virtual components.
PDSA cycle 2	Address persistent confusion about program structure; reinforce referral criteria and follow-up workflows; improve family understanding of the hybrid format.	Delivered additional staff education; incorporated portal access checks into rooming workflows; reinforced program expectations during visits; shifted to visit-by-visit scheduling based on family feedback.	SPC analysis demonstrated special-cause improvement in participation and treatment intensity, but not in referrals. Participation showed a clear upward centerline shift following implementation of PDSA cycle 2. Treatment intensity increased to a higher centerline with continued variation. Referrals increased compared with baseline but did not meet criteria for a sustained centerline shift.	Adopted visit-by-visit scheduling; strengthened in-visit referral and scheduling discussions; continued reinforcement of program expectations during clinical encounters.
PDSA cycle 3	Use PCORI-informed strategies to refine engagement; tailor communication based on patient characteristics and real-time data.	Reviewed demographic and clinical characteristics (language needs, comorbidities, BMI category); held staff discussions on engagement strategies; tailored outreach and messaging.	SPC charts demonstrated stabilization at higher performance levels. Participation remained above baseline with reduced variation, indicating improved process reliability. Treatment intensity stayed elevated with more consistent moving ranges. Referrals appeared modestly higher than baseline with reduced variability; however, criteria for a sustained centerline shift were not met.	Continued tailored communication strategies; refined timing, mode, and content of outreach to align with family priorities and sustain engagement.

**Fig. 2.**
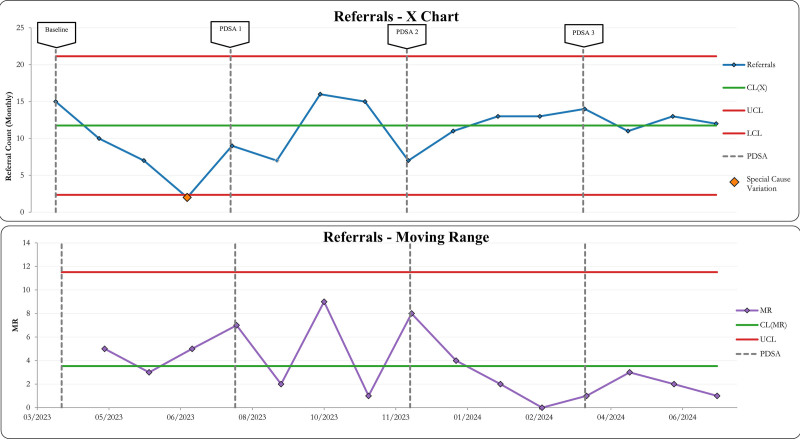
Referrals X-mR control chart. Individual monthly referral counts (X) and moving range (mR) are displayed. The centerline represents the process mean, and upper and lower control limits are shown at ±3 SDs. Special-cause variation was assessed using Provost criteria, including astronomical points beyond control limits and shifts defined as ≥6 consecutive points on one side of the baseline centerline. One astronomical point falls below the lower control limit; no sustained shift was identified.

**Fig. 3.**
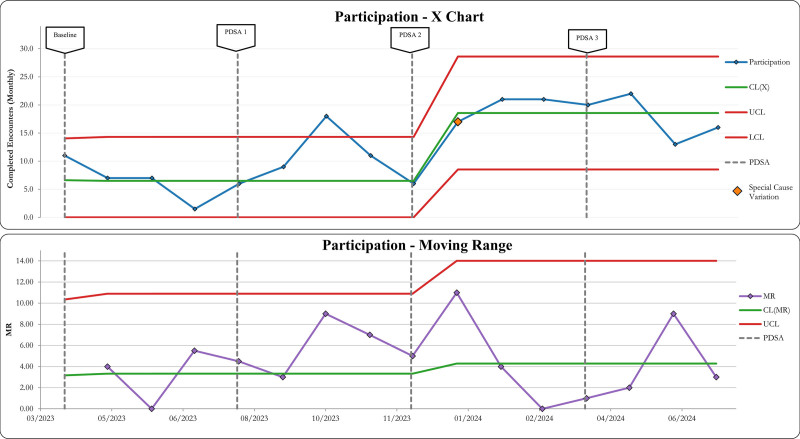
Participation X-mR control chart. Individual monthly participation counts (X) and moving range (mR) are shown. The centerline denotes the process mean, and control limits are calculated at ±3 SDs. Special-cause variation was evaluated using the Provost criteria, with a shift defined as ≥6 consecutive points above the baseline centerline. The first point of the shift is highlighted, and centerlines and control limits were recalculated to reflect new process performance.

**Fig. 4.**
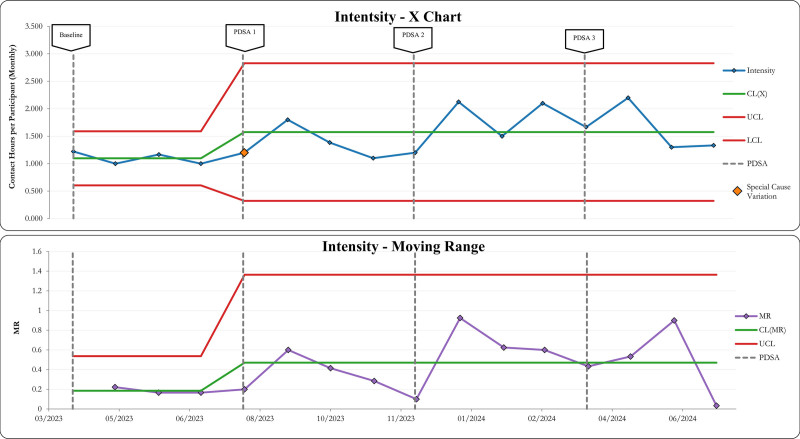
Intensity X-mR control chart. Monthly intensity values (X) and moving range (mR) are plotted with centerlines representing the process mean and control limits at ±3 SDs. Special-cause variation was assessed using the Provost criteria, which define a shift as ≥6 consecutive points above the baseline centerline. The first point of the shift is highlighted, and post-shift centerlines reflect updated process performance.

The table summarizes the aims, interventions, observed shifts in the SPC centerline, and resulting adaptations across three sequential PDSA cycles for implementing a hybrid IHBLT program. Outcomes are described using SPC centerline changes to align with the analytic approach used in Figures 2–4.

PDSA cycle 1 focused on formalizing the IHBLT program structure and clarifying expectations for staff and families. The team standardized the curriculum, developed culturally and linguistically appropriate educational materials, and implemented a hybrid care model combining in-person visits with technology-supported touchpoints. To support adoption, we delivered a focused provider education session during the monthly Faculty Education Meeting that introduced the program structure, eligibility criteria, and referral and scheduling workflows. Thirty-two clinicians attended or viewed the recording. A primary care–integrated referral algorithm for IHBLT is provided in **Supplemental Digital Content 1.** (**Figure 1, Supplemental Digital Content 1,** https://links.lww.com/PQ9/A770.) A sample patient recruitment flyer is available in **Supplemental Digital Content 2.** (**Table 1**, **Supplemental Digital Content 2,** https://links.lww.com/PQ9/A769.) The content was embedded as an electronic medical record (Epic Systems, Verona, Wis.) dot phrase and was accessible to referring providers for inclusion in the after-visit summary during well-child visits when referrals were initiated.

PDSA cycle 2 targeted persistent barriers in staff workflows and family understanding. We provided additional staff education on referral criteria, scheduling processes, and follow-up expectations. Rooming workflows were updated to include a portal access check, and IHBLT providers reinforced the program structure at each in-person visit. Based on family feedback that schedules changed frequently, we shifted from scheduling all three visits in advance to a visit-by-visit approach and strengthened in-visit scheduling conversations.

PDSA cycle 3 applied a patient-centered outcomes research institute–informed approach^18^ to refine engagement strategies. Staff reviewed real-time quality improvement data and examined patient characteristics, including language, comorbidities, and BMI category at the time of referral, to tailor outreach and messaging. We discussed engagement strategies during staff meetings and encouraged clinicians to align communication with family priorities and readiness for change.

### Study of the Interventions

The team used the model for improvement to link each PDSA cycle with explicit predictions and measures. We first conducted a retrospective review to establish baseline performance for referrals, participation, and contact hours. We then tracked these measures prospectively throughout implementation. Monthly performance data were displayed on statistical process control (SPC) charts and reviewed in team meetings to assess special-cause variation and inform subsequent cycles. Feedback from staff and families, gathered informally during visits and huddles, such as scheduling challenges and patient portal access difficulties, was integrated with quantitative data to refine interventions and address newly identified barriers.

### Measures

We selected three process measures to assess IHBLT adoption and treatment intensity: (1) program referrals, defined as the count of all referrals placed to the program; (2) program participation, defined as the count of completed Intensive Health Behavior and Lifestyle Treatment encounters; and (3) program intensity, defined as total contact hours per participant, including in-person encounters and virtual content. These measures reflected the program’s reach, engagement, and alignment with recommended treatment intensity. We did not include a formal balancing measure; however, the team monitored clinician workflow burden and scheduling capacity informally during implementation to ensure that interventions did not disrupt routine clinic operations.

### Analysis

We used SPC methods to assess changes in referral volume, program participation, and intervention intensity over time. Monthly data were plotted on control charts selected based on the measure type. Referral counts, participation, and intensity were displayed using appropriate individual-moving-range (X-mR) control charts, with accompanying moving-range charts, to assess process stability.

Baseline centerlines and control limits were established using preintervention data and held constant until special-cause variation was identified according to Provost criteria and SPC rules.^19^ Sustained shifts were defined as 6 or more consecutive data points above or below the baseline centerline. When sustained special-cause variation was identified, centerlines were recalculated to reflect new process performance.

Moving range charts were reviewed to assess short-term variation and confirm process stability. We used SPC analysis to support the interpretation of temporal changes associated with sequential PDSA cycles rather than to establish causal inference.

### Ethical Considerations

The Children’s Hospital Colorado Organizational Research Risk and Quality Improvement Review Panel reviewed this project and determined that it met the criteria for quality improvement and was exempt from further review. All activities were conducted to improve local care processes.

## RESULTS

To assess the impact of our interventions and identify performance trends, we analyzed the SPC charts (Figs. 2–4). This approach enabled us to visualize changes over time and evaluate the stability and effectiveness of the implemented strategies.

Referral volume remained largely stable throughout the study period and exhibited primarily common-cause variation. One isolated data point below the lower control limit occurred during the baseline period; however, no sustained shifts were observed following any PDSA cycle. These findings indicate no detectable change in the referral process over time.

In contrast, program participation increased following PDSA cycle 2 and met Provost criteria for special-cause variation, with 6 consecutive data points above the baseline centerline. This improvement was sustained over subsequent months, and centerlines were recalculated to reflect the new level of performance.

Intervention intensity demonstrated a similar sustained shift following PDSA cycle 2. We observed 6 consecutive points above the baseline centerline after implementation, indicating increased treatment exposure among participating families. Although some variability persisted, the higher intensity level was maintained across later phases.

Moving range charts demonstrated stable variation across all three measures, supporting the validity of observed centerline shifts. Overall, these findings suggest that system-level interventions were temporally associated with sustained improvements in participation and intervention intensity, whereas referral volume remained unchanged.

### Secondary, Exploratory Analysis: (Hypothesis-generating)

As a secondary, hypothesis‑generating analysis, we explored whether greater intensity, defined as total contact hours per participant, including in-person visits and virtual content, was associated with changes in BMI z-score (BMIz). After adjusting for age and sex, each additional hour of intervention exposure was associated with a 0.01 decrease in BMIz (*P* = 0.495). This association was not statistically significant. Given the project’s small sample size, nonrandom participation, and quality improvement focus, these findings were interpreted as exploratory rather than confirmatory.

## DISCUSSION

We implemented a hybrid IHBLT program in a busy academic primary care clinic, which improved participation and treatment intensity while maintaining stable referral volume. By standardizing curriculum content, tailoring educational materials, leveraging the patient portal, and applying sequential PDSA cycles, we increased program reach and delivered higher-intensity lifestyle treatment more reliably. The hybrid model is thought to have reduced logistical barriers, such as travel and scheduling, which commonly limit participation among families facing social and economic constraints.

### Interpretation

Our findings align with the broader evidence base showing that technology-supported IHBLT models can extend reach and feasibility. The sustained special-cause improvement in participation and program intensity suggests that the interventions led to a genuine system-level change rather than temporary fluctuations. The observed narrowing of intensity variation over time indicates that staff delivered higher-intensity care more consistently, reflecting improved system reliability. These findings suggest that improving system reliability and increasing IHBLT intensity are achievable and necessary precursors to improving patient‑level outcomes.

We observed an exploratory trend between intervention intensity and BMIz. Although the association did not reach statistical significance, it aligns with prior literature showing that greater treatment exposure is associated with improved weight-related outcomes.^1,2,4,5^ Notably, this pattern persisted when we increased contact hours using asynchronous virtual content. Because this project used a quality improvement design with a small, nonrandom sample, we interpreted these findings as hypothesis-generating rather than confirmatory. Future quality improvement initiatives or pragmatic trials that include BMIz as a secondary outcome could help clarify the relationship between hybrid IHBLT intensity and weight-related outcomes.

### Limitations

This project has several limitations. First, the program reach remained modest; referrals accounted for only a small fraction of eligible patients in the clinic, limiting the potential for population-level impact. Second, staff turnover and competing demands contributed to variability in referral patterns and may affect sustainability. Third, the small, nonrandom sample of participating families and the absence of a control group limit generalizability and preclude causal inference about patient-level outcomes. Finally, the exploratory BMI analysis was underpowered and should be interpreted cautiously.

## CONCLUSIONS

Primary care implementation of a hybrid IHBLT program is feasible and can improve referral patterns, participation, and treatment intensity for children with obesity. Our quality improvement approach, grounded in the model for improvement and supported by standardized materials and tailored engagement strategies, led to sustained gains in participation and treatment intensity, whereas referral volume remained stable. Although these findings are context-specific, the approach may be transferable to similar primary care settings facing workflow and equity constraints. Future work should focus on expanding reach, sustaining provider engagement, and rigorously evaluating patient-level outcomes, including changes in BMI and obesity-related comorbidities. Continued testing of tailored communication strategies and optimal timing of outreach may further enhance program accessibility and equity.

Our work builds on prior pediatric obesity quality improvement initiatives by integrating complementary strategies that have largely been tested in isolation. Earlier projects have demonstrated that standardizing provider workflows can improve referral and follow‑up ordering,^11,13^ whereas others have shown that redesigning care delivery through telemedicine can reduce access barriers and improve visit completion.^15^ In contrast, we focused on what happens after referral by standardizing program content and implementing a hybrid in‑person and technology‑supported IHBLT model within primary care. Rather than measuring success solely by referral volume or documentation, we evaluated participation and delivered treatment intensity, key prerequisites for clinical effectiveness. Using sequential PDSA cycles and SPC, we demonstrated sustained improvements in engagement and treatment dose despite stable referral rates. This approach advances the field by shifting the focus from prescribing weight management care to reliably delivering it, offering a pragmatic and scalable framework for primary care practices seeking to implement guideline‑concordant obesity treatment.

## ACKNOWLEDGMENTS

The authors thank Emily Petersen, Medical Librarian with the Health Literacy Program, and Carter Kasetani, Process Improvement Lead, both with the Children’s Hospital of Colorado, who aided with the study. Language editing assistance provided by Microsoft Copilot. The tool was used exclusively to improve clarity, grammar, and readability. No substantive content was generated or modified.

## Supplementary Material
