# Supplementary material for: Enhancing Pediatric Obesity Management through Quality Improvement: A Hybrid Approach to Intensive Health Behavior and Lifestyle Treatment in Primary Care
Source: Pediatr Qual Saf. 2026 Jul 28;11(4):e892. doi: 10.1097/pq9.0000000000000892 (PMC13412649; doi:10.1097/pq9.0000000000000892)
Supplement: Supplementary file 1 [file pqs-11-e892-s001.pdf]

SDC, Figure 1. Process Map for Primary Care Integrated IHBLT Referral

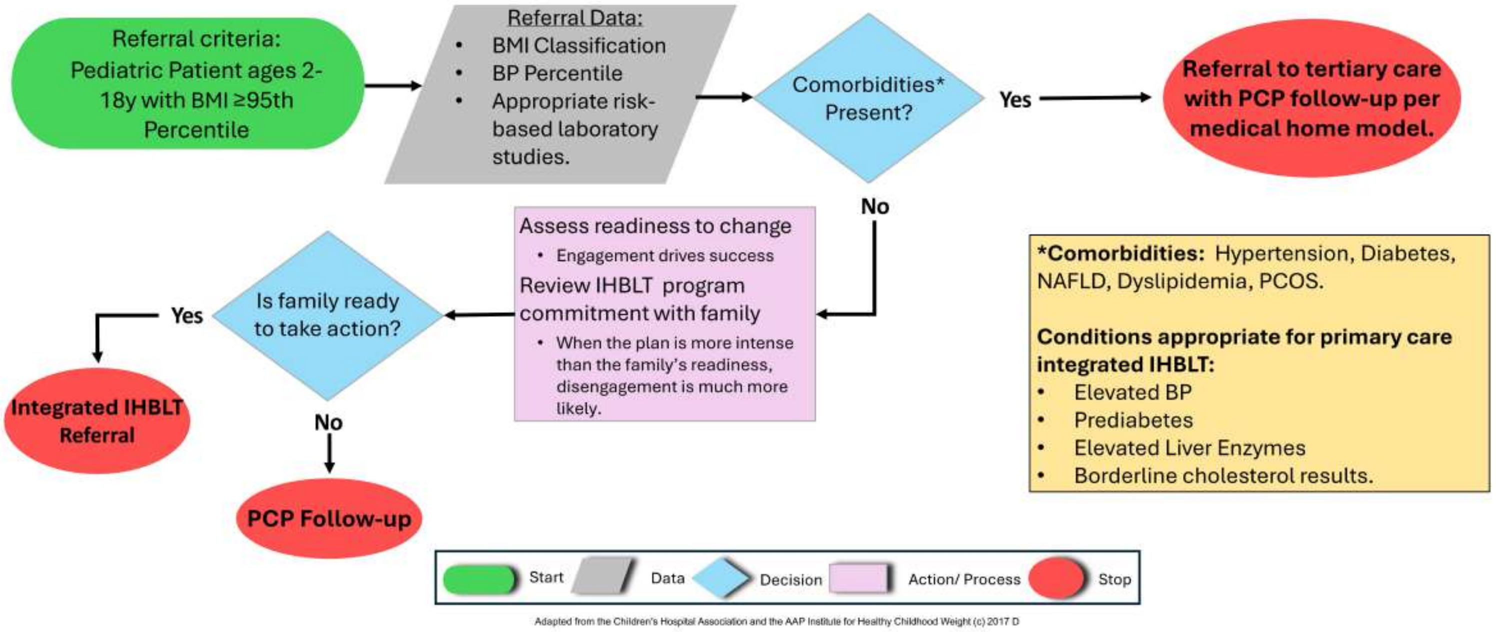

Figure Legend: Process map illustrating referral decision-making for Primary Care–Integrated Intensive Health Behavior and Lifestyle Treatment (IHBLT) among pediatric patients aged 2–18 years with BMI ≥95th percentile. The algorithm outlines screening criteria, assessment of obesity-related comorbidities, evaluation of family readiness to change, and referral pathways to either integrated IHBLT or tertiary specialty care with continued primary care follow-up. Comorbidities include hypertension, diabetes, nonalcoholic fatty liver disease (NAFLD), dyslipidemia, and polycystic ovary syndrome (PCOS). Adapted from the Children's Hospital Association and the AAP Institute for Healthy Childhood Weight (©2017).
