# Supplementary material for: Enhancing Pediatric Obesity Management through Quality Improvement: A Hybrid Approach to Intensive Health Behavior and Lifestyle Treatment in Primary Care
Source: Pediatr Qual Saf. 2026 Jul 28;11(4):e892. doi: 10.1097/pq9.0000000000000892 (PMC13412649; doi:10.1097/pq9.0000000000000892)
Supplement: Supplementary file 2 [file pqs-11-e892-s002.pdf]

SDC, Table 1. Overview of Intensive Health Behavior and Lifestyle Treatment (IHBLT)

What is Intensive Health Behavior and Lifestyle Treatment (IHBLT)?
Intensive Health Behavior and Lifestyle Treatment (IHBLT) is a safe and proven way to help children and teens who are overweight and have challenges with obesity.<br><br>IHBLT recognizes that each child has unique needs and can help both children and their families find strategies that work for them. IHBLT also helps families find ways around common barriers to healthy active living in a way that respects a family's cultural heritage and values.<br><br>Other names for IHBLT include *intensive behavioral intervention or family healthy weight programs.*
**The Primary Care Integrated IHBLT Program has:**<br>• 3 visits in person<br>• 9 messages sent through MyChart
**What we work on each month:**<br>• Month 1: Learning how to make healthy changes that last<br>• Month 2: Learning about healthy eating<br>• Month 3: Learning about moving your body and being active
**What to expect during IHBLT?**<br>The program offers ways to practice healthy behaviors and help you change behaviors in ways that work for your family:<br>• Content that focuses on health, not weight.<br>• Activities that focus on physical activity and healthy nutrition.<br>• Ways the whole household can help your child thrive in a healthy environment.<br>• A focus on changes that families can enjoy and keep up when IHBLT is over.<br>• Plenty of time! Changing routines and habits don't happen overnight. Our program has 12 weekly touchpoints over 3 months to help families succeed.
**What is the goal of IHBLT?**<br>The primary goals of IHBLT are:<br>• Overall good health<br>• Quality of life<br>• Self-esteem<br>• Respect for bodies of all shapes
**Do IHBLT programs work for kids of different ages?**<br>Yes. Anyone who takes part in IHBLT for at least 3 months should see improvements in health, fitness, nutrition habits, and quality of life.
